# PTEN Alterations as a Potential Mechanism for Tumor Cell Escape from PD-1/PD-L1 Inhibition

**DOI:** 10.3390/cancers11091318

**Published:** 2019-09-06

**Authors:** Daniele Cretella, Graziana Digiacomo, Elisa Giovannetti, Andrea Cavazzoni

**Affiliations:** 1 Department of Medicine and Surgery, University of Parma, 43126 Parma, Italy; 2 Department of Medical Oncology, Cancer Center Amsterdam, Amsterdam UMC, VU University Medical Center (VUmc), 1081HV Amsterdam, The Netherlands; 3 Fondazione Pisana per la Scienza, 56017 Pisa, Italy

**Keywords:** PTEN, PD-L1, immunotherapy, T-cells

## Abstract

The recent approval of immune checkpoint inhibitors drastically changed the standard treatments in many advanced cancer patients, but molecular changes within the tumor can prevent the activity of immunotherapy drugs. Thus, the introduction of the inhibitors of the immune checkpoint programmed death-1/programmed death ligand-1 (PD-1/PD-L1), should prompt deeper studies on resistance mechanisms, which can be caused by oncogenic mutations detected in cancer cells. *PTEN*, a tumor suppressor gene, dephosphorylates the lipid signaling intermediate PIP_3_ with inhibition of AKT activity, one of the main effectors of the PI3K signaling axis. As a consequence of genetic or epigenetic aberrations, PTEN expression is often altered, with increased activation of PI3K axis. Interestingly, some data confirmed that loss of PTEN expression modified the pattern of cytokine secretion creating an immune-suppressive microenvironment with increase of immune cell populations that can promote tumor progression. Moreover, PTEN loss may be ascribed to reduction of tumor infiltrating lymphocytes (TILs), which can explain the absence of activity of immune checkpoint inhibitors. This review describes the role of PTEN loss as a mechanism responsible for resistance to anti PD-1/PD-L1 treatment. Moreover, combinatorial strategies between PD-1/PD-L1 inhibitors and PI3K/AKT targeting drugs are proposed as a new strategy to overcome resistance to immune checkpoint inhibition.

## 1. Introduction

Accumulating evidence suggests that agents targeting immune checkpoints produce clinical benefit, including prolonged response and survival. In particular monoclonal antibodies (mAbs) as nivolumab, pembrolizumab, atezolizumab, avelumab, and durvalumab directed to the programmed death-1/programmed death ligand-1 (PD-1/PD-L1) immune checkpoint pathway proved significant efficacy as single arm, and represents a new therapeutic opportunity for many cancer patients. Nevertheless, as reported for melanoma patients, the effect of these mAbs is often restricted to a reduced percentage of patients, with lack of durable response as a consequence of the appearance of intrinsic or acquired resistance mechanisms [1,2].

Recently, mutations of JAK1-2 kinases have been proposed as alterations responsible for primary resistance in melanoma [3]. Moreover, in the last few years, multiple lines of evidence suggested a correlation between PTEN loss and lack of sensitivity to anti-PD-1/PD-L1 therapy.

This review focuses on this correlation as well as on the rationale of targeting deregulated PI3K/AKT/mTOR signaling, coupled with anti PD-1/PD-L1 agents.

## 2. PTEN Pathway

**Figure 1:**
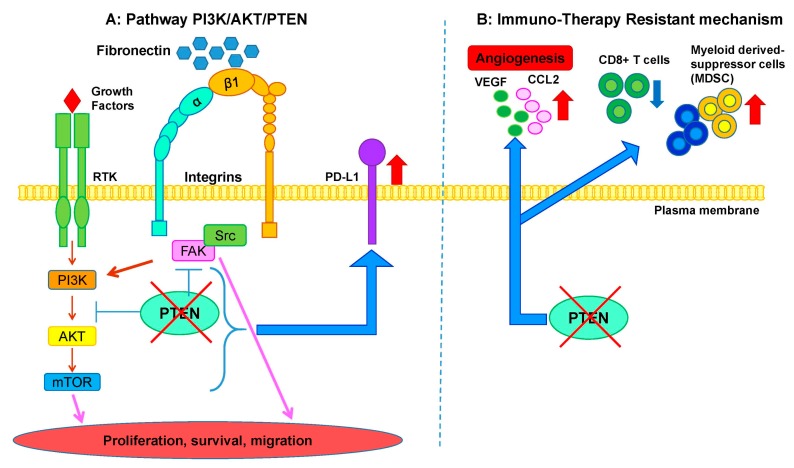
PTEN pathway and its implication in immuno-therapy resistance. (**A**) Overview of the PI3K/AKT/PTEN pathway. (**B**) Scheme of potential immuno-therapy resistance mechanisms.

The PI3K/AKT/mTOR axis is one of the main intracellular signaling pathways regulating a wide range of biological functions, such as cell growth and proliferation, cell energy metabolism, and expression of cytokines that regulate the immune compartment (Figure 1A). Membrane tyrosine Kinase Receptors (RTKs) activate phosphatidylinositol 3-kinase (PI3K) which in turn converts phosphatidylinositol (4,5)-bisphosphate (PIP2) in phosphatidylinositol (3,4,5)-trisphosphate (PIP3) [4]. PIP3 plays a key role as a second messenger, recruiting and activating AKT and PDK1 kinases at the plasma membrane. PDK1 phosphorylates AKT at the Thr308 residue and, in turn, activates other downstream effectors, including GSK3, FoxO, and the mTORC1 complex [5,6]. However, for the complete AKT activation, a further phosphorylation at the Ser473 residue is required: PIP3 by interacting with the pleckstrin homology (PH) domain of SIN1 [7], a component of mTORC2 complex, activates mTORC2 which in turn catalyzes AKT phosphorylation at Ser473 [6,7]. In addition, AKT can inhibit the tumor suppressive tuberous sclerosis complex (TSC1/2) [4] with consequent activation of the ras-like GTPase RHEB which in turn directly activates mTORC1 [8]. For its crucial role in controlling multiple intracellular processes, the PI3K-AKT-mTOR pathway is regulated by many negative regulators aiming at preventing an aberrant activation. One of the main mechanisms responsible for the constitutive activation of this pathway is the loss of phosphatase and tensin homolog deleted from chromosome 10 (PTEN) expression [9]. PTEN is a PIP3 3-phosphatase encoded by the *PTEN* gene located on chromosome 10q23 [10]. Structurally, the PTEN protein has an N-terminal PIP2-binding domain (PBD), a catalytic domain, a C2 lipidic domain, and a C-terminal domain containing a PEST (Pro, Glu, Ser, Thr) sequence [11,12]. Since PTEN is involved in the control of a wide range of processes including tumor growth and spread, metabolism, senescence and epithelial to mesenchymal transition (EMT), its downregulation plays a pivotal role in the progression of many types of cancer.

Moreover, alterations in the PI3K/AKT/mTOR pathway, e.g., PTEN loss, have an impact on cell energy metabolism and the metabolic reprogramming of cancer cells is another important hallmark of cancer. In particular, PTEN inactivation increased glucose uptake through the translocation of the glucose transporter 4 (GLUT4) protein at the plasma membrane [13]. The abrogation of PTEN function induces also the activation of 4EBP1 and p70S6 kinase, involved in the protein synthesis processes [14]. PTEN is involved in cell migration and cellular senescence as well. In particular, as reported for gastric and lung cancer, the down-regulation of PTEN expression is associated with the activation of the Focal Adhesion Kinase (FAK) which in turn leads to an increase of cell motility [15,16] (Figure 1A). It has been also described that PTEN inactivation may induce the loss of apical-basal polarity, promoting the EMT and consequently cellular dissemination [11,16]. Cellular senescence is an irreversible growth arrest process mainly controlled by p53, p21, and p16^ink4a^ proteins [17], and as reported for prostate cancer [18], PTEN loss can trigger cellular senescence both in vitro and in vivo.

## 3. PTEN Alterations

PTEN expression is not only controlled by the heterozygous or homozygous loss but also by a number of different molecular mechanisms, including epigenetic silencing, post-transcriptional and post-translational modifications, and protein-protein interactions. Starting from its localization at locus q23.3 of chromosome 10, an hotspot mutation site, the *PTEN* gene is susceptible to many alterations, such as point mutations, insertions, and deletions [9]. Since its identification, germline *PTEN* mutations have been frequently observed in a group of patients affected by a rare autosomal dominant syndrome note as PTEN hamartoma tumor syndrome (PHTS) [19]. In PHTS patients, these mutations are most frequently localized into the exon 5 of the *PTEN* gene, which encodes the phosphatase domain, but also exons 7 and 8 are affected [20]. Furthermore, somatic alterations occur in a wide range of cancers, including melanoma, glioblastoma, colon, breast, and lung cancer (Table 1) [21,22]. From ≈100,000 samples analyzed in the Catalogue of Somatic Mutation in Cancer (COSMIC) website, somatic alterations including point mutations, insertions, and deletions are reported in the whole *PTEN* gene. However, hotspot mutation sites have been identified at amino acids Arg130, Arg173, Arg233 among the phosphatase domain and the C2 domain (https://cancer.sanger.ac.uk/cosmic) [9,23]. Beside genetic alterations, PTEN expression can be regulated by epigenetic mechanisms as well. In fact, in melanoma and lung cancer the hypermethylation of CpG islands on the *PTEN* promoter induces the silencing of the *PTEN* gene [24,25]. Moreover, the *PTEN* promoter has different transcription factor binding sites, which may enable the regulation of PTEN expression. Among these, SNAIL, c-Jun, and NF-kB can downregulate PTEN expression [26,27,28,29]. Conversely, other transcription factors, such as p53, EGR1, and PPARγ can upregulate PTEN expression [30,31,32]. In particular, several lines of evidence have underlined how PTEN and p53 can regulate each other: by binding the *PTEN* promoter at its responsive element (RE), p53 induces PTEN expression and, on the other hand, PTEN indirectly increases p53 level by reducing MDM2 expression [33]. In the last decade, microRNAs (miRNAs) emerged for their role as regulators of gene expression at the post-transcriptional level. These 22 nucleotides RNAs caused mRNA degradation when fully paired to the seed region, binding site at the 3′ untranslated region (3′ UTR) of the mRNA target. The miR-17-92 cluster has been associated with downregulation of PTEN in lymphoproliferative diseases [34]. The oncomir miR-21 is frequently altered in different tumors, including lung, ovarian, and hepatocellular cancer. miR-21 can downregulate PTEN expression by directly targeting its 3′UTR and therefore reducing PTEN mRNA translation (Table 1) [16,35]. Different other miRNAs can regulate PTEN expression in other cancers, including miR-19a, 22, and 25 (Table 1). The fine regulation of PTEN expression is also affected by post-translational modifications such as phosphorylation, acetylation, oxidation, and ubiquitination. Concerning the role of PTEN phosphorylation as a mechanism of post-translational modification, six phosphorylation sites in the C-terminal domain have been reported for PTEN [36]. These sites are mainly phosphorylated by SRC, CK2, and GSK3β kinases resulting in a deregulation of PTEN function [37,38,39]. PTEN activity is also downregulated by acetylation through the histone acetyltransferase P300/CBP-associated factor (PCAF) and by oxidation through the interaction with reactive oxygen species (ROS) [40,41]. The reduction of PTEN expression can be a consequence of its degradation as well. Recent evidence identified neural precursor cell expressed developmentally down-regulated protein 4–1 (NEDD4–1), an E3 ubiquitin protein that can target PTEN causing its degradation by the proteasome system [42]. The PTEN deregulation by the ubiquitination is one of the main mechanisms involved in many types of cancers, including NSCLC [43]. Recent evidence demonstrated that protein-protein interactions are able to regulate PTEN activity affecting its conformation, stability, and localization. For example, the interaction with membrane-associated guanylate kinase inverted 2 (MAGI2) can induce PTEN activity. On the other hand, Parkinson Protein 7 (PARK7) binds PTEN under oxidative stress conditions with a consequent inhibition of PTEN function [44]. 

## 4. PTEN Loss can Modify the Tumor Microenvironment

### 4.1. PTEN Loss and Alteration of T-Cell Activity in Melanoma Patients

Several lines of evidence have underlined the role of PTEN loss on melanoma progression and resistance to immunotherapy management in patients sequentially treated with anti-Cytotoxic T-Lymphocyte Antigen 4 (CTLA-4) and anti PD-1 [63] agents. A high burden of copy number loss was reported in non-responsive patients: further analysis revealed that copy number loss was recurrent in 9p and 10q chromosome regions. Among these areas, *PTEN* maps in the 10q region. Finally, the authors reported a correlation between PTEN loss and resistance to immunotherapy treatment, suggesting that deletion of the *PTEN* gene might be one of the driver resistance mechanisms exploited by tumors to PD-1/PD-L1 inhibition.

The 30% of melanoma patients with PTEN loss often presented a V600E BRAF point mutation [64,65]. The presence of both alterations is responsible for development of metastatic lesions in a mouse model [51] and the inhibition of both signaling (BRAF and PI3K/AKT) caused a regression of melanoma, but upon arrest of drug administration, melanoma restart to grow indicating the presence of long-lived initiating cells. In this context PTEN abrogation could identify a different subset of melanoma patients: recently, it has been reported that PTEN loss promotes resistance to immunotherapy in a melanoma preclinical model [66] (Figure 1B). Stable silencing of the *PTEN* gene in human and murine melanoma cancer cells significantly increased the expression of immunosuppressive cytokines, resulting in decreased T-cell infiltration, and inhibition of autophagy, which reduced the killing properties of T-cells both in in vitro and in vivo systems. Interestingly, the effect of the anti PD-1 pembrolizumab was less highlighted in patients with PTEN-absent tumors as a consequence of reduced TILs in the tumor microenvironment. Similarly, the authors reported that in stage IIIB/C melanoma patients, the loss of PTEN expression is significantly correlated with low CD8+ tumor infiltration, in respect to tumors with high PTEN level. In the small percentage of patients with heterogeneous PTEN expression, the regions with low PTEN levels are associated with low TILs infiltration, compared to regions with high PTEN level [66].

Further analysis of the melanoma TCGA data set revealed that reduction of PTEN copy number was significantly associated with low expression of IFN-ϒ mRNA, a classical signature of T-cell activation. Moreover, the frequency of PTEN mutations or deletions is high in non-inflamed tumors [66].

Interestingly, in melanoma cancer cells and patients, loss of PTEN function does not correlate with PD-L1 expression, enforcing the lack of relevance of PD-L1 in this context. The authors identified other immune-regulatory factors mainly up-regulated by PTEN loss, in particular two cytokines, CCL2 and VEGF, known to induce immune suppression in the tumor microenvironment [66,67] (Figure 1B). Extensive analysis of inflamed-related genes demonstrated a significant reduction of their expression, suggesting that in melanoma, loss of PTEN contributed to the reduction of tumor inflammation. 

### 4.2. PTEN Loss and Tumor Microenvironment Modification in Glioblastoma

The glioblastoma is one of the most aggressive malignancies of the brain. At present, the current standard of care has reduced efficacy, with an average overall survival of 16–20 months [68], and the anti PD-1 treatment showed efficacy only in a small percentage of patients (8%) [69]. A correlation between PTEN and immunotherapy resistance has been observed [70], where cytotoxic activity of CD8+ T-cells was more pronounced toward glioma cells wild type for PTEN. Recently, Zhao and coworkers reported that in a cohort of 32 patients non-responsive to immunotherapy [71], 23 patients presented PTEN mutations, whereas only three patients in the responsive group reported PTEN alterations, confirming that PTEN changes are preferentially detected in non-responders. Moreover, as expected, high activity of the PI3K/AKT pathway was described in the tumors of non-responders, but without difference of PD-L1 expression between responders and non-responders, suggesting that PTEN did not correlate with PD-L1 expression, at least in this tumor type. With the aim to further explore the mechanism responsible for the immune resistance, the authors profiled the immune infiltrate and observed a correlation between PTEN mutation and high levels of macrophages, microglia, and neutrophils in the tumor microenvironment, reporting a correlation between PTEN mutation and the tumor infiltrate. Tumor associated macrophages (TAMs) often act as protumoral macrophages, contributing to disease progression by promoting tumor spread and metastasis, inhibition of cytotoxic T-cell activity, and by secreting some cytokines to favor neo-angiogenesis and tumor progression [72]. These results underline the ability of PTEN to modulate the tumor microenvironment, probably as a consequence of altered pattern of secreted cytokines in the tumor stroma.

### 4.3. PTEN Loss and Tumor Senescent Phenotype in Prostate Cancer

Prostate cancer is the second most common cause of male cancer-related death worldwide. A recent study described the relevance of oncogenic alterations of PI3K axis by the discovery of point mutations in the *PI3KCA* isoform [73] that contribute to cancer progression. In particular, these genetic mutations often overlap with one of the most significant genetic aberrations, the loss of the *PTEN* gene, estimated in 40%–50% of patients with prostate cancer [74].

As reported for melanoma and glioblastoma, PTEN alterations in prostate tumors contribute to altering the tumor microenvironment [75]. Interestingly, the authors reported that PTEN null mice developed a tumor-senescent phenotype, associated with a massive secretion of immunosuppressive cytokines, as a consequence of the activation of JAK2/STAT3 pathway in cancer cells. In order to investigate the role of STAT3 on immune suppressive cytokine production, the authors used an engineered mouse model knockout for both *PTEN* and *STAT3* genes. In line with their findings, a significant reduction of immune suppressive cytokines was reported. Moreover, the phenotype of tumor-infiltrating immune cells meaningfully changed: in particular, a more relevant percentage of NK, CD8+, and B cells was reported, associated with a marked decrease of myeloid-derived suppressor cells (MDSC) and a reduction of markers of senescence in the tumor. Finally, tumors null for *PTEN* and *STAT3* were smaller in size, with significant reduction of tumor stroma. As docetaxel is the gold standard first line treatment for hormone-refractory prostate tumors, the authors observed that single treatment with the anti-microtubule agent improved tumor reduction marginally in PTEN null cancers, confirming data from clinical trials [76]; if docetaxel is coupled with a specific JAK inhibitor, a dramatic shrinkage of the tumor was observed, as a consequence of the reprogrammed cancer phenotype to an inflamed tumor, with reduction of immune suppressive cytokines and accumulation of pro-inflamed stromal cells in the tumor microenvironment.

At the molecular level, PTEN null cells showed a significant downregulation of SHP2, a negative controller of the JAK/STAT3 pathway. It was stated that loss of SHP2 protein sustains tumor growth by promoting JAK/STAT3 pathway activation. This correlation was further validated for other tumor types [77,78]. 

### 4.4. PTEN Loss and Modification of T-Cell Function in Leiomyosarcoma

To date the response to immune targeted agents is reported for some solid tumors, in particular carcinomas and melanoma, but there is poor data from tumors of mesenchymal origin [79]. Recently, a case of leiomyosarcoma was reported [80]. After 2 years of anti-PD-1 treatment, the patient presented no sign of malignancy, except a low, but continuous growing local metastasis. The molecular analysis of this tumor revealed focal amplification of the *myc* oncogene and a large heterologous deletion of the chromosome region containing the *PTEN* gene in the pre-treatment tumor. The other *PTEN* allele presented a missense mutation in the exon coding the catalytic domain in the tumor resistant to treatment. Interestingly, a VEGF increase was reported in this treatment-resistant tumor, as previously reported for melanoma [66]. Extended analysis of sarcomas from the TCGA archive and matched for both (*MYC* and *PTEN*) molecular alterations detected in this patient, revealed a constitutive STAT3 expression (absent in MYC amplified tumors), as also identified in prostate tumors [75]. Parallelly, a significant decrease of PD-1+ T-cells was observed in the microenvironment of treatment-resistant tumor, as a consequence of a global reduction of CD8+ T-cells in the tumor infiltrate.

Globally, the loss of PTEN, associated with a reduction of T-cell function and cellularity as well as with increased VEGF production, identifies a common tumor phenotype similar to previous reported data in melanoma, prostate, and glioblastoma tumors.

### 4.5. PTEN Loss in Lung Carcinoma

Melanoma and lung cancer are two tumors currently treated with immune checkpoint inhibitors directed to PD-1/PD-L1 or CTLA-4. Considering that PTEN is often mutated in lung cancers [4,81], with a percentage close to 15% in the squamous histology [82], recent studies have evaluated if PTEN inactivation contributes to squamous lung cancer progression. Recently, in [83] it was reported that, in a mouse model, concomitant bi-allelic loss of *PTEN* and *LKB1* contributed to development of lung cancer with squamous histology. Interestingly, a significant enrichment in tumor associated neutrophils (TANs) was observed in the tumor microenvironment of these animals.

The high percentage of TANs is a consequence of the secretion of chemoattractant chemokines (CCL1, 2, 5, and G-CSF) by tumor epithelial cells, that in turn recruit and activate TANs [84], strictly involved in tumor spread and metastasis [84].

To further examine the immune infiltrate in these tumors, a marked increase of T-reg cells compared to T-CD8+ population is documented. Moreover, in the T-cell fraction, high PD-1 membrane levels and high IL-6 and TGF-β cytokine concentration were reported in the tumor microenvironment. Both cytokines have been stated to promote immune evasion and surveillance, with consequent increased spread of tumor cells and metastasis [85,86]. Additional data exploring the immune phenotype in this context revealed an increase of PD-L1 in tumor cells, indicating that bi-allelic loss of *LKB1* and *PTEN* can significantly induce suppression of activity of CD8+ T-cells.

Concerning the correlation between PTEN and PD-L1 in lung cancer, conflicting results have been proposed [87,88]. Interestingly, Hlaing and coworkers observed a positive correlation between PTEN expression and PD-L1 level, differently from the previously reported data for other tumor types, with a 80% of correlation observed in the squamous histology [88]. Currently, a rational explanation is not provided, but the authors speculated on the mechanisms responsible for PD-L1 upregulation and arrest of immune surveillance in the tumor microenvironment. PD-L1 can be directly regulated by intracellular signaling pathways, mainly deregulated in many tumor types (RAS/RAF/MEK, PI3K/AKT/mTOR, JAK, and STAT) or by the IFN-ϒ signaling, as a consequence of IFN-ϒ release by immune cells in the tumor microenvironment. High PD-L1 expression in patients with high PTEN levels was probably a consequence of high IFN-ϒ produced in these tumor types. By this way, PTEN positive tumors can evade immune surveillance by increasing PD-L1 protein levels. These data are partially confirmed by a recent case report [89], where the presence of *PTEN* and *LKB1* alterations were reported in a NSCLC patient in advanced stage. Additionally, high PD-L1 expression was reported, confirming previous observations. Interestingly, this patient was not responsive to immune checkpoint inhibition, in spite of high PD-L1 and tumor mutational burden (TMB), but surprisingly it showed an exceptional response to an mTOR inhibitor. The lack of durable response to immune checkpoint inhibition in the presence of high PD-L1 expression and TMB, associated with the success of targeting specific genomic alterations suggested that in a PTEN loss context, further assessment and investigation are required.

## 5. Correlation between PTEN and PD-L1

The role of PTEN as “supervisor” of the response to PD-1/PD-L1 inhibitors is not only restricted to changes in the tumor microenvironment, but some data reported the ability of PTEN to modulate PD-L1 level. As reported in a seminal article [90], loss of PTEN or constitutive expression of the PI3K/AKT pathway can regulate PD-L1 expression in some tumor types in both IFN-Y dependent and independent manners.

The PI3K/AKT signaling is mainly activated by interferons and controls the mRNA translation of interferon-dependent genes. By pharmacological inhibition with PI3K or AKT targeting agents, a significant reduction of IFN-ϒ-induced PD-L1 is reported [90]. 

The PI3K axis can also regulate PD-L1 expression independently from IFN-ϒ release, as reported for many solid tumors. For instance, the inhibition of PI3K/AKT/mTOR pathway with specific targeting agents reduced PD-L1 levels in NSCLC cells; in particular the authors of this study reported that PD-L1 expression is dependent from mTOR signaling and then pharmacological inhibition of mTOR by rapamycin or AZD8055 markedly reduced PD-L1 level [91].

The increase of PD-L1 by constitutive activation of PI3K signaling is at least in part ascribed to altered PD-L1 mRNA levels [90]. Moreover, PI3K signaling is also involved in the up-regulation of PD-L1 by extracellular stimuli (i.e., EGF in NSCLC cells, and 17β-estradiol in estrogen-receptor-α positive breast cancer cells [90]).

In colorectal cancer cell lines [92], silencing of PTEN caused PD-L1 increase at the membrane level, without increase of PD-L1 mRNA, suggesting that PTEN loss could contribute to PD-L1 protein stabilization. Interestingly, IFN-ϒ does not affect the ability of PTEN to modulate PD-L1, revealing a different mechanism of regulation. In colorectal patients, the authors reported a relationship between PTEN loss and PD-L1 expression, but only a partial correlation with distant metastasis, TNM, and overall survival. Moreover, a strong correlation between PD-L1 and TNM and metastasis was reported. These data suggest that the connection between PD-L1 and cancer progression is only a marginal consequence of PTEN loss. A similar relationship was reported for gastric cancer [93]: in particular, patients with PTEN loss of heterozygosity (LOH) displayed higher PD-L1 expression. On the contrary, this correlation was not observed in mesothelioma patients [94].

## 6. Discussions

Deregulated expression of the *PTEN* gene is often observed in solid tumors, and recently it was associated with resistance to immune checkpoint inhibition. To date, loss of PTEN expression is only marginally correlated with up-regulation of PD-L1 on the membrane surface of tumor cells; in fact, PTEN abrogation often caused alteration in the tumor microenvironment, with increase of a non-inflamed tumor as a consequence of the release of anti-inflammatory cytokines and significant reduction of T-cell activity.

The presence of infiltrated T-cells in the tumor microenvironment is associated with high percentage of immunotherapy efficacy, including targeting of the immune checkpoint PD1/PD-L1. Considering the critical role exerted by T-cells, it is relevant to know the mechanisms that cause T-cell accumulation or reduction, in order to develop or employ new strategies for restoring the T-cell population in the tumor microenvironment. Several mechanisms are proposed as candidates for resistance to immune checkpoint inhibition: one of the most important mechanisms is the constitutive activation of the PI3K/AKT/mTOR signaling axis, as a consequence of the activation of upstream oncogenic drivers, such as NRAS, KRAS, and RET [91]. Moreover, loss of PTEN function is an alteration responsible for constitutive activation of this signaling, as a consequence of point mutation, gene deletion, or hyper-methylation events [23]. This pathway is both active in tumor cells and in the microenvironment, regulating not only cancer growth and dissemination, but also the activity of immune cells inside the tumor and other processes as neovascularization and reorganization of tumor matrix [95]. 

Recently, it has been demonstrated that the microenvironment can alter the gene expression profile of tumor cells. In particular, disseminated tumor cells, often acquire essential traits from the metastatic microenvironment for successful growth of tumors; in this context the peculiar characteristics of cells of the microenvironment can significantly modify the RNA expression signature of disseminated tumor cells. As reported [96], loss of PTEN expression is relevant in brain metastasis [97,98] as a consequence of the release of exosomal miRNA targeting PTEN mRNA in cancer cells by resident astrocytes. This evidence supports the hypothesis that metastatic sites are different, based on the niche where metastasis grows, and the knowledge of metastatic lesions could provide new anti-metastatic and personalized therapies. 

In this context, further studies should re-examine of specific targeted agents directed to the PI3K/AKT/mTOR pathway, that failed in monotherapy on the majority of solid tumors [99], as single treatment, whereas in a combination regimen a partial response (PR) was reported, compared to single-agent therapy [100]. This may be due to intrinsic or acquired resistance to PI3K inhibition, or the consequential stimulation of parallel intracellular signaling pathways activated in tumor cells after PI3K inhibition [101]. In particular, in a PTEN loss context, increased activity of the PI3K-β isoform [102] and the efficacy of PI3K-β isoform inhibition, by specific targeted agents (GSK2636771, AZD8186) [103], could overcome the resistance mechanisms to immune targeted agents, as reported for example in melanoma patients [66]. Since PI3K is involved in T-cell activation as well, the optimization of the schedule regimens may be considered; otherwise employing specific PI3K isoform inhibitors (not relevant for T-cell activity, i.e., PI3K-β) without anti-tumor activity and sparing of T-cell function could be taken into consideration. It is also important to consider that PI3K-α and β isoforms are predominantly expressed in non-immune cells; on the contrary, PI3K-ϒ or δ isoforms largely control PI3K pathway activation in immune cells (T-cells, myeloid derived suppressor cells, and T-regs); because of these findings, the use of agents specifically directed to PI3K α and β isoforms, which are mainly detected in the tumor cells, can produce only marginal side effects on TILs and other immune cells in the tumor microenvironment [95,104].

However, accumulation of T-reg cells in the tumor microenvironment is a crucial process in the immune evasion, as a consequence of cytokines released by tumor and stromal cells that recruit T-regs at the tumor stroma. T-reg-mediated immunosuppression causes a relevant reduction of CD8+ and CD4+ functionality in the tumor stroma and can recruit myeloid-derived suppressor cells. In this context the inhibition of PI3K-ϒ can reprogram T-regs and myeloid cells from an immunosuppressive to an immune-stimulatory phenotype, restoring the number of functional CD8+ cells that can synergize with immune checkpoint inhibitors.

Recent studies reported several emerging mechanisms of resistance to immune checkpoint inhibition. Among these mechanisms, myeloid cells have a major role in limiting effective tumor immunity [105]. In particular an interesting study showed that high infiltration of tumor associated myeloid cells induced resistance to immune checkpoint blockade; while selective pharmacologic targeting of PI3K-ϒ, highly expressed in myeloid cells, restored the sensitivity to immune check point agents by switching the tumor microenvironment to a pro-inflammatory phenotype [106]. Similar results have been proposed in a head and neck cancer model [107].

Moreover, the PI3K axis exerts a critical role in other cells of the tumor stroma; in particular, the α isoform has been shown to be the most important isoform in endothelial cells, in both blood and lymph vessels [108,109]. Some pre-clinical studies performed with pan-class I PI3K inhibitors showed reduced total intra-tumor vessel area with reduced vessel function [110] but these effects were mild when compared to standard anti-angiogenic therapy. Currently, only a few data describe the role of the PI3K axis in the fibroblasts associated with tumor stroma. In a breast cancer model [111] functional PTEN inactivation has been reported in cancer-associated fibroblasts, contributing to cancer development and progression. Although the effects of specific PI3K inhibitors have not been reported, the tumor-promoting activity by cancer-associated fibroblasts might therefore be inhibited by employing PI3K targeting agents.

Different drugs are currently in clinical trials for the treatment of cancer patients with loss of PTEN. Several of these clinical studies are evaluating the benefit of PI3K/AKT/mTOR inhibition in association with immune checkpoint inhibitors in patients with colorectal, lung, leukemia, or other solid tumors (Table 2).

Additional data focusing on the rationale combination of PI3K inhibitors and anti PD-1/PD-L1 agents is reported for breast cancer [112]. In a preclinical in vivo model of murine breast cancer, the effect of concomitant BKM120 (a pan-PI3K inhibitor) and anti PD-1 treatment is better than the effect of single agents. Interestingly, the authors reported that BKM120 does not alter the number of CD8+ T-cells inside the tumor, but an increase of CD4+ T-cells migrating on the neoplastic lesion was observed, with production of IFN-ϒ. Moreover, the differentiation of myeloid cells to the monocyte lineage is stimulated by BKM120, enforcing the pro-inflammatory properties as a consequence of PI3K inhibition. Keeping with these data, PI3K inhibitors showed a pro-inflammatory role, with massive IFN-ϒ secretion [113]. 

Loss of PTEN function is not only correlated with constitutive PI3K/AKT signaling activation but also with enforcing the intracellular activity of the focal adhesion kinase FAK [16,114] and concomitant inhibition of both signaling is necessary to obtain synergistic effects on tumor cells [16]. To date, accumulating data implementing the role of FAK as an enzyme involved in the regulation of the immune compartment of tumor microenvironment are reported [115]. The authors described that the nuclear form of FAK is involved in the transcriptional activation of some cytokines, responsible for alteration of the immune context inside the tumor. In particular, the authors reported massive CCL5 and TGF-β production in a squamous cell carcinoma (SCC) tumor model, that is reverted by FAK kinase inhibition. Both CCL5 and TGF-β can alter the immune context by blocking CD8+ T-cell activity and stimulating the T-reg subpopulation with the generation of an immune suppressive phenotype in the tumor microenvironment. Further results enforced the role of FAK as a critical kinase involved in the generation of an anti-tumor immune evasion setting in a murine model of SCC [116].

Currently, new clinical trials designed to target the PI3K aberrant activation in tumors, coupled with immune checkpoint inhibition are under evaluation. In this context, it is important to take into consideration the mechanisms of acquired resistance to immune checkpoint inhibition. As reported [117], late relapses are now emerging in patients treated with anti PD-1/PD-L1 therapy. To date, considering the few data concerning the resistance mechanisms to immune check point inhibition in different tumor types, we can only speculate that PTEN loss should be considered a potential biomarker for the combined treatment with PD-1/PD-L1 targeting agents and PI3K inhibitors. However, based on data reported in Table 2, some clinical trials proposed to select the patients based on PI3K/AKT/PTEN alterations. For example, the NCT03772561 study includes the evaluation of the relationship between AKT/PIK3CA/PTEN mutations and response. In the NCT03842228 study, the phosphorylation of AKT, S6, 4EBP1, three members of PI3K/AKT/mTOR signaling, will be correlated with response to treatment. In the NCT03131908 study the patients were selected based on PTEN loss by immunohistochemistry (IHC) or molecular analysis. Finally, in the clinical trial NCT03673787 the patients were selected based on pathogenic mutations in PIK3CA, AKT1, AKT2, identified by next generation sequencing or PTEN loss by IHC.

## 7. Conclusions

Genetic re-analysis of lesions after initial response of tumor cells could reveal new acquired tumor mutations that might affect immune-evasion mechanisms. Therefore, while the effect of specific agents targeted to these genetic lesions does not show any significant effect in terms of tumor shrinkage, it does cause an effect on the tumor microenvironment. This effect, especially on T-cell function, should prompt further studies on new therapeutic strategies to re-activate immune cells, combined with immune checkpoint targeted agents. 

## Figures and Tables

**Table 1:** Major structural genetic alterations of PTEN gene in solid tumors.

Tumor Type	Mutation (%)	Deletion (%)	LOH (%)	Promoter Methylation (%)	miRNA Alteration	Reference
Breast cancer	3–5	20–40	30–40	30–50	miR-106b, -93 up-regulation	[45,46,47]
Lung cancer	6–9 predominantly in SQCLC	20–40	20–40	20–30	miR-21 up-regulation	[10,22,24,45,48]
Colorectal cancer	10–20	5–15	10–20	10–20	miR-21, -32 up-regulation	[10,22,49,50]
Melanoma	10–20	5–10	20–40	30–50	miR-25, -221, -222 up-regulation	[10,22,25,28,51]
Glioma	30–40	40–70	50–70	5–10	miR-26a up-regulation	[10,22,52,53]
Prostate cancer	5–15	10–20	20–50	1–5	miR-22 up-regulation	[10,22,54,55]
Ovarian	1–5	30–40	30–50	5–10	miR-19a, -21, -214 up-regulation	[10,22,56,57,58]
Pancreatic cancer	<1	15–20	30–50	<1	miR-21 up-regulation	[10,22,59,60]
Kidney cancer	<1	1–5	20–30	<1	miR-23b-5p up-regulation	[10,22,61,62]

SQCLC: squamous cells lung carcinoma; deletion: loss of both alleles; LOH: loss of heterozygosity.

**Table 2:** Clinical trials with PI3K targeting agents coupled with PD-1/PD-L1 inhibitors (http://clinicaltrials.gov/).

Agent	Target	in Combination with	Tumors	Phase	Reference
Capivasertib (AZD5363)	AKT	Olaparib + Durvalumab	Advanced solid tumors	I	NCT03772561
Copanlisib (BAY80–6946)	PI3K	Nivolumab	Colorectal	I/II	NCT03711058
		Olaparib + Durvalumab	Solid tumors	1b	NCT03842228
		Nivolumab	Solid tumors and lymphomas	1b	NCT03502733
IPI-549	PI3K-γ	Atezolizumab + Nab-Paclitaxel Atezolizumab + Bevacizumab	Breast cancer Renal cell carcinoma	II	NCT03961698
		Nivolumab	Solid tumors	I	NCT02637531
GSK2636771	PI3K-β	Pembrolizumab	Melanoma	I/II	NCT03131908
Ipatasertib (RG7440)	AKT	Atezolizumab	Solid tumors	I	NCT03673787
		Atezolizumab	Breast cancer	1b	NCT03800836
Idelalisib (GS-1101)	PI3K-δ	Pembrolizumab	Lung	1b/2	NCT03257722
SF1126	PI3 Kinase and Bromodomain Inhibitor	Nivolumab	HCC	I	NCT03059147
Tenalisib (RP6530)	PI3K-δ/γ	Pembrolizumab	cHL	I/II	NCT03471351
Copanlisib (BAY80–6946)	PI3K	Nivolumab	Colorectal	I/II	NCT03711058
		Olaparib + Durvalumab	Solid tumors	1b	NCT03842228
		Nivolumab	Solid tumors and lymphomas	1b	NCT03502733

cHL (classical Hodgkin Lymphoma), HCC (hepatocellular carcinoma).
